# Alternative Splicing of Neuropeptide Prohormone and Receptor Genes Associated with Pain Sensitivity Was Detected with Zero-Inflated Models

**DOI:** 10.3390/biomedicines10040877

**Published:** 2022-04-10

**Authors:** Bruce R. Southey, Sandra L. Rodriguez-Zas

**Affiliations:** 1 Department of Animal Sciences, University of Illinois at Urbana-Champaign, Urbana, IL 61801, USA; rodrgzzs@illinois.edu; 2 Department of Statistics, University of Illinois at Urbana-Champaign, Urbana, IL 61801, USA

**Keywords:** migraine, nitroglycerin, transcript isoform, trigeminal ganglia, nucleus accumbens, alternative splicing, neuropeptide, zero inflation

## Abstract

Migraine is often accompanied by exacerbated sensitivity to stimuli and pain associated with alternative splicing of genes in signaling pathways. Complementary analyses of alternative splicing of neuropeptide prohormone and receptor genes involved in cell–cell communication in the trigeminal ganglia and nucleus accumbens regions of mice presenting nitroglycerin-elicited hypersensitivity and control mice were conducted. De novo sequence assembly detected 540 isoforms from 168 neuropeptide prohormone and receptor genes. A zero-inflated negative binomial model that accommodates for potential excess of zero isoform counts enabled the detection of 27, 202, and 12 differentially expressed isoforms associated with hypersensitivity, regions, and the interaction between hypersensitivity and regions, respectively. Skipped exons and alternative 3′ splice sites were the most frequent splicing events detected in the genes studied. Significant differential splicing associated with hypersensitivity was identified in CALCA and VGF neuropeptide prohormone genes and ADCYAP1R1, CRHR2, and IGF1R neuropeptide receptor genes. The prevalent region effect on differential isoform levels (202 isoforms) and alternative splicing (82 events) were consistent with the distinct splicing known to differentiate central nervous structures. Our findings highlight the changes in alternative splicing in neuropeptide prohormone and receptor genes associated with hypersensitivity to pain and the necessity to target isoform profiles for enhanced understanding and treatment of associated disorders such as migraine.

## 1. Introduction

Migraines are a debilitating condition afflicting approximately 30% of the world population. Migraine pain is frequently accompanied by intracranial hypersensitivity to stimuli and central sensitization or allodynia [1,2]. Migraineurs frequently report cephalic and extracephalic allodynia, including hypersensitivity to stimuli and pain (hyperalgesia), and tenderness in the head and extremities [3]. Advances in understanding the molecular pathways underlying hypersensitivity to pain have been gained from studying the expression profile of genes as a whole [4]. This study of gene expression in the trigeminal ganglia and nucleus accumbens of mice presenting cephalic and extremity hypersensitivity to pain elicited by nitroglycerin (NTG) identified significant differences in 275 genes relative to the control. The previous genes were annotated to the circadian rhythm and glutamatergic and dopaminergic synapse processes, including prohormone genes in signaling pathways that code for neuropeptides such as proenkephalin (PENK) and natriuretic peptide type C (NPPC), and neuropeptide-like protein chromosome 4 open reading frame (C4orf48) [4]. Reports of the role of neuropeptides such as pituitary adenylate-cyclase-activating polypeptide (PACAP) and receptors in migraine [5,6,7] and retinal and ocular disorders [5,8,9,10,11] emphasize the participation of these signaling molecules across central nervous system regions.

Despite the attention to gene pathways and profiles, less attention has been directed to understanding the relationship between hyperalgesia and the individual transcript isoforms resulting from alternative splicing. Evidence is accumulating about the relationship between alternative splicing products and migraine, pointing to new insights into the disease. For example, splice variation in calcium channel pore-forming α1-subunit gene has been associated with familial hemiplegic migraine [12], and sodium voltage-gated channel alpha subunit 1 (SCN1A) has been associated with migraine without aura [13]. Two recent reviews have highlighted the role of the calcitonin gene-related peptide (CGRP) on migraines and effective treatments [14,15]. The neuropeptide CGRP results from cleavage of a calcitonin-related polypeptide alpha (CALCA) isoform, but bioactive neuropeptides resulting from other CALCA isoforms including calcitonin are not known to participate in migraine. Two of the isoforms produced by the alternative splicing of the gene cocaine- and amphetamine-regulated transcript protein (CARTPT) are translated into bioactive peptides [16] involved in pain signaling processing [17].

Many neuropeptide prohormone genes such as CALCA, CARTPT, and tachykinin precursor 1 (TAC1) undergo alternative splicing, with the resulting neuropeptide products having different functions. The levels of alternative splicing isoforms from 38 neuropeptide prohormone and receptor genes were affected by maternal immune activation in pig hippocampus and amygdala [18]. The impacted isoforms included peptide YY (PYY), calcitonin-related polypeptide beta (CALCB), relaxin 2 (RLN2), secretogranin II (SCG2), neuropeptide Y receptor Y5 (NPY5R), glucagon-like peptide 1 receptor (GLP1R), and oxidized low-density lipoprotein receptor 1 (OLR1) [18]. The previous alternative splicing events may impact migraine and pain because maternal immune activation during gestation has been associated with autism and schizophrenia spectrum disorders that present a high incidence of migraine and pain sensitivity comorbidities [19,20].

Although there is ample evidence supporting the association between hypersensitivity to pain and neuropeptide signal–receptor systems, the participation of gene isoforms is limited. This situation is partly due to the small size of many neuropeptide isoforms, resulting in skewed detection distribution. Therefore, standard models have limited statistical power to test for differential abundance or splicing, even in experiments with adequate sequencing depth. Accurate testing of differential neuropeptide isoform levels necessitates non-standard zero-inflated models [21,22].

With this study, we aim to advance the understanding of changes in alternative splicing processes and events in neuropeptide prohormone and receptor genes associated with hypersensitivity to pain. Sequenced reads from an RNA-seq experiment that profiled two central nervous system regions from mice presenting hypersensitivity to pain and controls were used. The standard gene-centric expression study [4] was superseded using de novo assembly and prediction methods to reconstruct all transcript isoforms in the samples. Zero-inflated models were used to identify neuropeptide prohormone and receptor transcript isoforms with differential expression due to hyperalgesia. Alternative splicing events of neuropeptide prohormone and receptor genes associated with hyperalgesia were identified. The combination of de novo assembly, differential transcript expression, and differential splicing events were used to identify alternative splicing of neuropeptide and receptor genes that underly hyperalgesia phenotypes.

## 2. Materials and Methods

### 2.1. Animals Experiments, Sample Extraction, and Sequencing

The animal experiment [4] used C57BL6/J male mice (Jackson Laboratories, Bar Harbor, ME, USA) between 9 and 12 weeks of age housed in groups under a 12/12 dark/light regime with food and water made available ad libitum. Mice were randomly allocated to a group experiencing migraine-like behaviors elicited by chronic nitroglycerin or a control group. The established protocol to elicit hyperalgesia or hypersensitivity to pain stimuli involved intraperitoneal nitroglycerin injections (10 mg/kg diluted in saline at 0.9%) over 9 days, alternating injections between days [4,23]. The mode of action of NTG is by donating nitric oxide, a vasodilator that triggers headaches [24,25]. Nitroglycerin is used in rodent models of migraines because, relative to controls, mice subjected to the nitroglycerin protocol (NTG group) have a higher sensitivity to mechanical pain in cephalic and extremity regions and other migraine-related behaviors [4,23,26,27]. Control mice (CON group) were intraperitoneally administered saline in a volume equal to the nitroglycerin and saline dose received by the first group. Mice in the NTG group presented higher (*p*-value < 0.0001) mechanical sensitivity to noxious stimuli elicited by von Frey filaments applied to the cephalic region and hind paw separately [4].

On the tenth day, one day after the final injection, the mice received the anesthetic pentobarbital (Somnosol, Henry Schein Animal Heath, Dublin OH, USA), were euthanized, and were promptly intracardially injected with cold saline to maximize preservation. Two central nervous regions associated with sensation and mediation of pain, the trigeminal ganglia (TG) and the nucleus accumbens (NAc), were extracted from each mouse [28,29]. The previous regions were snap-frozen and preserved at −80 °C, followed by tissue homogenization, and nucleotides were extracted using an RNA kit (Omega Biotek, Norcross, GA, USA).

The set of 20 RNA samples across the two treatment groups and regions had high RNA quality (integrity > 7.5). The individual samples were paired-end sequenced using HiSeq 4000 (Illumina, San Diego, CA, USA), providing sequences of 100 nucleotides long with a mean Phred quality score >30 that were subsequently analyzed. The gene-level analysis of these samples was published [4], and the FASTQ files, including the RNA sequences, are available from the National Center for Biotechnology Information (NCBI) Gene Expression Omnibus repository (GEO) [30], experiment identifier GSE110194. The alternative splicing analysis pursued in this study necessitated a distinct de novo approach for transcript isoform identification and a unique model suited for the spliced isoform abundances and profiles.

### 2.2. De Novo Transcript Isoform Identification

The transcript isoforms in the individual samples were predicted using the de novo assembly approach implemented in the software Trinity (v2.12) with default settings [31,32]. Subsequently, the isoform predictions were searched using the BLAST (v2.10.1) routines [33] with default settings, except the low-complexity filter was disabled. Isoform predictions were searched in a database that included the NCBI RefSeq [34] mouse sequences corresponding to the 95 neuropeptide prohormone and 84 neuropeptide receptor genes to maximize identifications [35]. Sequence matches with complete sequence identity and no gaps were classified as complete, matches with at least 95% sequence identity and no gaps were classified as partial, and the remaining sequences were classified as unmatched. Neuropeptide prohormone and receptor gene sequence matches were then summarized within treatment and region across samples according to the frequency of detection across samples, combined treatment–region group, and the extent of the sequence match (i.e., complete, partial, or none).

De novo predicted neuropeptide prohormone protein sequences were compared to neuropeptides detected in several rodent peptidomics studies. The peptidomics information corresponded to multiple central nervous regions, including the amygdala [36], dorsal horn [6], habenula [37], hippocampus [36,38], hypothalamus [6,36,38,39], NAc [6,38], periaqueductal gray [6], prefrontal cortex [36], rostroventral medulla [6], suprachiasmatic nucleus [40,41,42], supraoptic nucleus [43], striatum [36] and dorsal striatum [38], thalamus [36], TG [6], and trigeminal nucleus [6].

### 2.3. Differential Expression of Spliced Products

The individual samples were mapped to transcript isoforms from the mouse genome (NCBI mm39) using the software Salmon (v1.4.0) [44] with the validateMappings option, and other settings were set to default values. The number of reads mapped to transcript isoforms of known neuropeptide prohormone and receptor genes was analyzed using a model suited to the count distribution across isoforms resulting from alternative splicing. The zero-inflated negative binomial model was used to describe the isoform read counts. This model combines a negative binomial distribution and a logit distribution and accommodates any overdispersion for isoform zero counts that exceed the assumptions. This overdispersion associated with the misalignment between observed and predicted zero counts correspond to “sampling zeros” and “structural zeros” [21,22]. Sampling zeros arise from sampling variation where a gene or transcript isoform was not detected but may have been present. Structural zeros occur when genes or transcript isoforms are not expressed in all samples [45]. Standard models to analyze gene or isoform expression levels rely on incompatible assumptions when excessive zero counts are present. The zero-inflated negative binomial model enabled tests for treatment, region, and interaction effects on the isoform levels while addressing excessive zero counts. The model was implemented using the GENMOD procedure in the SAS software (SAS Systems Inc., Cary, NC). The *p*-values of the model effects were adjusted for multiple testing using the False Discovery Rate (FDR) adjustment [46].

### 2.4. Differential Alternative Splicing

The study of differences in alternative splicing modalities between treatment and region groups required the use of alignment and testing approaches distinct from those used for testing differential isoform abundance. Individual samples were aligned to the NCBI mm39 mouse genome assembly using the software STAR with ENCODE standard options [47]. The differential alternative splicing between NTG and CON groups and between regions were tested using the software rMATS v4.1.1 [48] with a specification of 100 for readLength and default values for the remaining parameters. Pairwise comparisons between treatments were conducted across all samples and within each region, and a similar strategy was used for region comparison. The alternative splicing mechanism, including skipped exon, mutually exclusive exons, alternative 3′ splice site, alternative 5′ splice site, and retained intron, were identified and counted using reads that span joins (Junction Counts) and reads that did not cross an exon boundary (Exon Counts).

## 3. Results

### 3.1. De Novo Sequence Prediction

De novo assembly of sequence reads from an RNA-seq experiment enabled the detection and subsequent profiling of isoforms from neuropeptide prohormone and receptor genes associated with hypersensitivity to pain across two central nervous system regions. The depth and coverage of the RNA-seq experiment are characterized by 140,000,000 reads/sample for an approximate total of 2800 billion nucleotides across the 20 samples studied. Although the mouse transcriptome was analyzed in the present study, the mouse genome is 2.5 billion nucleotides long for reference.

The curated 94 mouse neuropeptide prohormone genes encompassed 494 NCBI annotated isoforms originating from alternative splicing. Overall, de novo protein predictions were mapped to 85 neuropeptide prohormone genes with 231 transcript isoforms at various levels of completeness. The distribution of the number of neuropeptide prohormone and receptor protein sequences detected in the NTG and CON groups across regions is listed in Table S1. Only protein sequences from 23 neuropeptide prohormone genes were detected in all mouse samples, and 59 neuropeptide prohormone genes had partial matches in both regions and treatments. Neuropeptide prohormone genes detected in all samples included CARTPT, neurotensin (NTS), platelet-derived growth factor B polypeptide (PDGFB), SCG2, secretogranin III (SCG3), and TAC1. Neuropeptide prohormone genes were detected in all samples, although the protein sequences were partially recovered in some samples, including insulin-like growth factor 1 (IGF1), vasoactive intestinal polypeptide (VIP), KiSS-1 metastasis-suppressor (KISS1), and prokineticin 2 (PROK2). The partial detection of some neuropeptide prohormone genes may reflect transitional processes because 19 neuropeptide prohormone protein sequences were recovered entirely in at least one sample. Some isoforms from the partially recovered gene sequences are expected to present zero counts, and zero-inflated models are apt to describe these cases.

The de novo prediction was validated by the 40 neuropeptide prohormone genes, including annotated peptides in the NCBI database, and 21 neuropeptide prohormone genes also detected in 10 or more peptide experiments. Table S2 summarizes the neuropeptide genes obtained by de novo assembly that were also detected in 10 peptidomics experiments. The de novo predictions were validated by the detection of 20 out of the 21 neuropeptide prohormone genes in 70% to 100% of the samples. All de novo predictions were consistent with sequences detected in the previous peptidomics experiments. While tachykinin precursor 3 (TAC3, also known as TAC2) was detected in 55% of the samples, tachykinin family co-member TAC1 was detected in 100% of the samples.

Parallel to the neuropeptide prohormone gene analysis, 84 mouse neuropeptide receptor genes encompassing 635 NCBI RefSeq annotated isoforms resulting from alternative splicing were studied. Overall, 83 neuropeptide receptor genes with 309 transcript isoforms were detected in the samples with different levels of completeness from the de novo predictions (Table S1). Among the detected neuropeptide receptor genes, 9 genes were recovered entirely in all samples, 33 genes were recovered entirely in at least one combined region and treatment group, and the sequence of 25 genes was partially recovered across regions and treatments. The detection of neuropeptide receptor genes was more variable between regions than treatments. Among the neuropeptide receptor genes detected in either region, 19 genes, including parathyroid hormone 2 receptor (PTH2R), relaxin/insulin-like family peptide receptor 1 (RXFP1), and relaxin/insulin-like family peptide receptor 2 (RXFP2) were detected exclusively in the NAc, and OLR1 and gonadotropin-releasing hormone receptor (GNRHR) were detected exclusively in the TG.

### 3.2. Differential Transcript Isoform Expression

Among the curated list of neuropeptide prohormone and receptor mouse genes, nine genes were not analyzed due to insufficient reads. Across all samples, no reads were assigned to the gastric inhibitory polypeptide (GIP), insulin I (INS1), and PYY, and reads from pancreatic polypeptide (PPY), prolactin-releasing hormone (PRLH), parathyroid hormone (PTH), urotensin 2 (UTS2), insulin II (INS2), and arginine vasopressin receptor 2 (AVPR2) were only detected in less than three samples. This read distribution resulted in 202 neuropeptide prohormone transcript isoforms analyzed from 87 neuropeptide prohormone genes and 249 neuropeptide receptor transcript isoforms analyzed from 83 neuropeptide receptor genes. No transcript isoform exhibited a significant excess of zero read counts after FDR adjustment.

Only 12 transcript isoforms exhibited a significant (FDR-adjusted *p*-value < 0.10) interaction between treatment and region (Table 1). This interaction was driven by a large difference between regions, leading to a significant difference between regions for all transcript isoforms. A smaller difference between treatments was observed with only five transcript isoforms with a significant (FDR-adjusted *p*-value < 0.10) difference between treatments. However, the single transcript (NM_053093.2) of tachykinin precursor 4 (TAC4) was an exception, as NTG in TG was significantly different from the other treatments and regions.

The 11 transcript isoforms that exhibited a significant (FDR-adjusted *p*-value < 0.05) expression differential between treatments and a significant (FDR-adjusted *p*-value < 0.05) expression differential between regions did not exhibit a significant interaction between treatment and region (Table 2). Except for two transcript isoforms, these transcripts were underexpressed in NAc compared to TG.

There were nine transcript isoforms from seven neuropeptide prohormone genes and seven transcript isoforms from five neuropeptide receptor genes that only exhibited significant (FDR-adjusted *p*-value < 0.10) differential abundance between treatments (Table 3). While the transcript isoforms from CALCA, gastric inhibitory polypeptide receptor (GIPR), and insulin-like growth factor 2 (IGF2) had a similar pattern, the platelet derived growth factor, alpha (PDGFA), and PTH2R transcript isoforms had a differing response.

The 202 transcript isoforms that exhibited significant (FDR adjusted *p*-value < 0.1) region differences were approximately evenly distributed between overexpressed and underexpressed in NAc compared to TG (118 compared to 84). Genes with at least three transcript isoforms (Table 4) generally exhibited similar fold changes between transcript isoforms. However, six genes had one transcript isoform with a different direction than the other transcript isoforms. No differences in overall gene expression between regions were detected for calcitonin receptor-like (CALCRL) and PDGFA because the expression levels of the multiple isoforms coded by either gene canceled out.

### 3.3. Alternative Splicing

#### 3.3.1. Type of Alternative Splicing Events Detected

There were 149 splicing events observed from 27 neuropeptide prohormone genes and 249 splicing events observed from 31 neuropeptide receptor genes. The prevalence of the different types of splicing events was similar between neuropeptide prohormone and receptor genes, such that skipped exons represented the most common alternative splicing event detected (58% of all events and 33% of all genes), followed by alternative 3′ splice site (14% of all events and 18% of all genes) and mutually excluded exons (13% of all events and 9% of all genes).

#### 3.3.2. Differential between Treatment

Significantly different (FDR-adjusted *p*-value < 0.1) treatment splicing events were detected in two neuropeptide prohormone and three neuropeptide receptor genes (Table 5). Splicing events in neuropeptide prohormone genes occurred in the 5′ untranslated region and were overabundant in NTG compared to CON. The CALCA events were rarely detected in NAc (41 reads from nine samples) compared to TG (21,707 reads from 10 samples). In both cases, the same splicing event was rarely detected in the other region with a consistent pattern (< 10% of all reads assigned to each splicing event). The two events of ADCYAP receptor type I (ADCYAP1R1, also known as PAC1) refer to the same skipped exon because an alternative 3′ splice site event occurs with the skipped exon. The same differential splicing event was detected for corticotropin-releasing hormone receptor 2 (CRHR2) across regions, but after adjustment for multiple testing, the difference was not significant in the NAc (unadjusted *p*-value < 0.005). An insulin-like growth factor 1 receptor (IGF1R) skipped exon was detected in both regions, but the number of reads adjusted by exon length was only significant in NAc.

#### 3.3.3. Differential between Regions

There were 82 significant alternative splicing events (FDR-adjusted *p*-value < 0.1) neuropeptide prohormone (24) and receptor (58) genes between regions. The most frequent alternative splicing events were skipped exons (39%), mutually exclusive exons (24%), and alternative 5′ splice sites (21%). The frequent neuropeptide prohormone genes were VGF nerve growth factor inducible (VGF) (5 events) and SCG3 (4 events), and the frequent receptor genes were opioid receptor-like 1 (OPRL1) (17 events), ADCYAP1R1 (8 events), and natriuretic peptide receptor 2 (NPR2) (7 events). Across all three comparisons, 24 significant (FDR-adjusted *p*-value < 0.1) alternative splicing events from 12 genes were observed (Table 6). Most alternative splicing events involved the same region and were associated with known transcript isoforms.

## 4. Discussion

Insights into the participation of alternative splicing in neuropeptide prohormone and receptor genes on hypersensitivity to pain, a condition frequently experienced by migraineurs, were gained by advanced isoform analysis of transcriptome profiles [4]. De novo assembly of the reads from an RNA-seq study of the TG and NAc enabled thorough detection of all the isoforms across samples. Detection was followed by testing for differential isoform levels between treatment and region groups using zero-inflation models and differential splicing events. The range of alternative splicing mechanisms detected in the present study offers insights into the variety of processes by which hyperalgesia conditions could regulate gene expression and neuropeptide or receptor production.

Differential alternative splicing events associated with hyperalgesia in the untranslated regions (UTRs) were detected in neuropeptide prohormone and receptor genes with different transcription start sites or non-coding exons. Alternative splice sites involving the UTR impact the stability, translational efficiency, and localization of the mRNA [49], suggesting additional ways by which mRNA gene expression is modulated in hyperalgesia conditions. Differential splicing in the UTR of VGF, a single-exon coding gene detected in the present study, was supported by the de novo assembly recovery of the complete mRNA sequence in all 20 samples. Moreover, VGF transcript isoforms exhibited significantly higher expression in NAc than TG. The association between VGF splicing and hyperalgesia is consistent with reports that VGF tends to be overexpressed in pain signaling structures of many neuropathic pain animal models [50]. Reports of elements that regulate the expression of VGF through action in the promoter and single exon could relate to the mutually excluded non-coding exon detected in the present study [51].

Other types of differential alternative splicing events associated with hyperalgesia encompassed changes in the translated mRNA sequence resulting in new neuropeptides or the absence of known neuropeptides. The impact of nucleobindin 2 (NUCB2), SCG2, and SCG3 alternative splicing events identified by rMATS are unknown, but the SCG2 events would remove an experimentally uncharacterized neuropeptide. Neuropeptide prohormone genes TAC1, CALCA, and IGF1 and neuropeptide receptor genes ADCYAP1R1, CRHR2, and IGF1R presented alternative splicing events, and changes in the resulting isoforms may impact the presence and severity of hyperalgesia.

**Figure 1:**
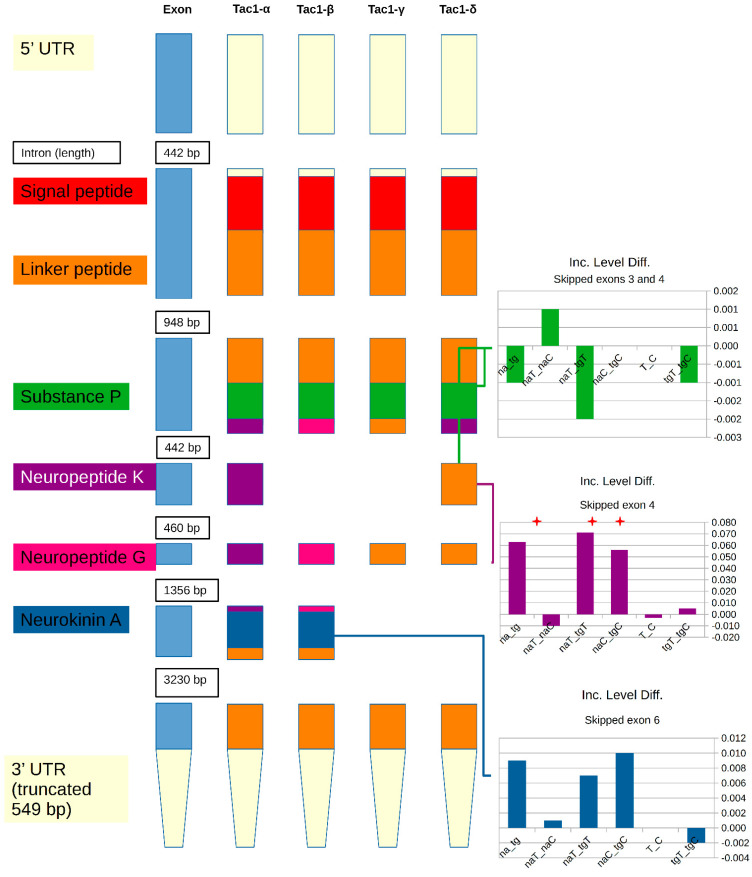
Schematic representation of tachykinin precursor 1 gene illustrating exons and the neuropeptides produced from the known transcript isoforms resulting from skipped exon events. Inclusion level differences (Inc. Level Diff.) between the inclusion and exclusion of the corresponding exon are provided for different comparisons involving region (na = nucleus accumbens; tg = trigeminal ganglia) and treatment (T = nitroglycerin-treated; C = control). Within the label, the underscore separates the combinations of regions and treatments used in each comparison. Significant (FDR-adjusted *p*-value < 0.1) differences are denoted by the red star.

The alternative splicing of TAC1 resulted in four mutually exclusive transcript isoforms that were also identified in de novo assembly. While only two transcript isoforms are currently annotated in the mouse assembly, these transcript isoforms correspond to human and rat α-, β-, γ-, and δ-TAC1 isoforms [52]. A schematic representation of the Tac1 gene (Figure 1) illustrates the differences between transcript isoforms and peptide production of the neuropeptides neuropeptide K, neuropeptide gamma, and neurokinin A. The predominant alternative splicing events detected by rMATS were the skipping of exons 4 and 6. An additional rare event (<1% of assigned reads) corresponded to skipped exons 3 and 4. The inclusive level difference indicates a higher inclusion of exons 4 and 6 in NAc than in TG (Figure 1), for example, a 0.6 inclusive level difference was observed between NAc and TG (denoted as na_tg in Figure 1). While all four protein isoforms produce substance *p*, Figure 1 reveals that neuropeptide K is produced only by β-TAC1, neuropeptide gamma is only produced by γ-TAC1, and neurokinin A can be produced from both β-TAC1 and γ-TAC1. The α-TAC1 and δ-TAC1 isoforms have truncated peptide sequences of neuropeptide K and neuropeptide gamma, respectively, that lack the C-terminal neurokinin A sequence and associated cleavage site. Among the genes presenting mutually exclusive events in the NAc and TG, neurokinin A tends to be associated with migraine and pain [53], while neuropeptide K tends to be associated with hypotension [52].

The known alternative splicing events in CALCA correspond to mutually exclusive exons and result in different protein isoforms that contain the neuropeptides CGRP and calcitonin. While de novo assembly identified the CGRP-containing transcript isoform, a possible calcitonin transcript isoform was identified. This transcript included an intron that would provide the calcitonin transcript isoform when spliced. Transcripts for both protein isoforms and the CALCR were underexpressed in NTG compared to CON, and the calcitonin producing isoform was underexpressed in NAc compared to TG. The significant alternative splicing of CALCA between NTG and CON detected by rMATS corresponded to alternative 3′ and 5′ splice site events in the 5′ untranslated region of a non-coding exon. Aligned with our finding of differential splicing in the 5′ untranslated region of CALCA, a single nucleotide polymorphism (rs3781719) located in the promoter region of CALCA has been associated with the response of patients to the OnabotulinumtoxinA chronic migraine therapy [54].

IGF1 has 20 transcript isoforms arising from different alternative start sites and alternative terminal regions that can be translated into 15 unique protein sequences. Differential expression between hyperalgesia and control groups was identified in the IGF1 isoforms encompassing the terminal region. The association between IGF1 isoforms and hyperalgesia detected in this study is supported by reports that the administration of IGF1 is proven effective in alleviating sensory neuropathy [55]. Notably, more unique IGF1 protein sequences (NP_001104745.1 and NP_001300939.1) were encoded by the isoforms in the TG than in the NAc. Associations between spliced products of the IGF1 and sensitivity to stimuli comorbidities have been reported. An isoform of IGF1 was differentially expressed in the hippocampus between pigs exposed to maternal immune activation during gestation and controls [18]. Maternal immune activation has been associated with behavioral disorders, including autism and schizophrenia spectrum disorders and sensitivity to stimuli [20].

Isoforms coded by the receptor ADCYAP1R1 exhibited significant differential expression and alternative splicing events between the hyperalgesia and control groups. Differential expression of ADCYAP1R1 transcript isoforms in the pig hippocampus was associated with maternal immune activation [18]. Two almost identical skipped exon events in ADCYAP1R1 corresponded to the hip and hop exons [56,57], and the differentially expressed isoform lacked the hop exon. The association between nitroglycerin-induced hypersensitivity to pain and splicing events detected in the present study could be related to the requirement of the pituitary adenylate cyclase-activating polypeptide for the development of spinal sensitization and establishment of neuropathic pain [58]. The activity of ADCYAP1R1 can be fine-tuned through alternative splicing variants that modify the ligand binding and signaling properties of this receptor [57].

All de novo predicted neuropeptide prohormone gene isoforms were consistent with corresponding peptides experimentally identified in 10 other neuropeptidomic studies. The de novo assembly presented in this study enabled the detection of 250 isoforms from 75 neuropeptide genes and 323 isoforms from 83 receptor genes. In addition, de novo predicted previously undiscovered transcript isoforms in AUGN, chromogranin A (CHGA), and TAC1. The novel AUGN isoform involved an alternative 3′ splice site and would result in a truncated augin neuropeptide. The novel CHGA isoform involved a skipped exon and would result in a truncated beta-granin neuropeptide. This information can be used in neuropeptidomic studies to find novel peptides; for example, information from de novo transcripts was used to detect neuropeptides in nudipleuran gastropods by mass spectrometry [59].

Differential alternative 3′ splice site splicing events in CRHR2 between hyperalgesia and control groups were observed in the TG and across regions. The CRHR2 splicing event involves modifications in the α1 exon comparable with the products of different initiation sites between CRHR2 isoforms [60]. The changes in the alternative splicing of CRHR2 could be related to abnormalities in CRHR2 correlated with pain and the use of CRHR2 antagonists against chronic pain [61]. Differential alternative 3′ splice site splice events were detected in IGF1R between the hyperalgesia and control groups within the NAc. The splicing events in IGF1R associated with hyperalgesia could correspond to the effect of IGF1R inhibition on reducing thermal and mechanical pain hypersensitivity [62]. Altogether, these results highlight a range of alternative splicing and isoform expression processes that can modulate the levels of neuropeptide prohormone and receptor mRNA that participate in hypersensitivity to pain.

The significant differences between hyperalgesia and control mice detected for isoforms in other genes are aligned with reports of the association between these genes and pain-signaling phenotypes. Both the neuropeptide prohormone gene and an associated receptor gene were differentially expressed. Opioid peptides from PENK and prepronociceptin (PNOC) and opioid receptor delta 1 (OPRD1) are associated with pain [63,64]. Neuropeptide FF-amide peptide precursor (NPFF) is associated with migraine, and the NPFF receptor, neuropeptide FF receptor 2 (NPFFR2), has a role in the modulation of nociception [65]. NPPC and an associated receptor, natriuretic peptide receptor 3 (NPR3), are associated with inflammation [66].

Neuropeptides directly associated with pain or migraine from significantly differentially expressed neuropeptide prohormone genes include apelin (APLN) [67,68], cortistatin (CORT) [69], IGF2 [70,71], neuromedin B (NMB) [72], neuropeptide W (NPW) [73], PDGFA and platelet-derived growth factor, D polypeptide (PDGFD) [74], TAC4 [75], and VIP [76]. The role of some neuropeptide prohormone genes may be indirectly associated with migraines or pain. Pro-melanin concentrating hormone (PMCH) has been associated with the influence of eating and glucose on migraines [77]. Natriuretic peptide type B (NPPB) and natriuretic peptide type C (NPPC) [78] and SCG3 [79] may act via the cardiovascular and neurovascular regulation.

Receptors directly associated with migraine or pain include endothelin receptor type B (EDNRB) [80], GIPR [81], and hypocretin receptor 1 (HCRTR1) [82]. Other receptors with possible indirect effects on migraine are parathyroid hormone 1 receptor (PTH1R) and PTH2R, which mediates calcium and phosphate homeostasis [83], and NPY5R that acts as receptor for neuropeptide Y (NPY), PYY, and PYY, and influences eating [84,85].

## 5. Conclusions

Different methodologies were undertaken to elucidate changes in alternative splicing processes in neuropeptide prohormone and receptor genes in a mouse model of hyperalgesia. Combining de novo assembly, transcript isoform analysis, and splice site variation provides an enhanced study of the neuropeptide prohormone and receptor complement. Model testing confirmed that zero-inflated modeling was well suited to describe the distribution of transcript isoforms within and across central nervous system regions.

A survey of alternative splicing events indicated that alternative 5′ splice site and skipped exons were the most frequent mechanisms to generate isoforms among the genes studied. The results from this study highlight the multiple modes of alternative splicing action that can regulate the expression of neuropeptide and receptor genes underlying hyperalgesia phenotypes, such as those endured by migraineurs. Understanding the role of this alternative splicing will provide the foundation for identifying isoform-based therapies to ameliorate hypersensitivity to migraine and pain.

## Figures and Tables

**Table 1:** Neuropeptide prohormone and receptor transcript isoforms and genes that present significant (FDR-adjusted *p*-value < 0.05) effect of nitroglycerin-elicited hypersensitivity to pain in a region-dependent (treatment-by-region) manner.

Symbol	Accession	FDR_PV ^1^		Treatment		Region
		FDR_PV	Dif ^2^	FDR_PV	Dif	
Prohormone						
APLN	NM_013912.4	6.67 × 10^−2^	3.77 × 10^−2^	NTG	1.79 × 10^−12^	NA
NPPC	NM_010933.5	2.48 × 10^−2^	2.59 × 10^−2^	NTG	4.78 × 10^−7^	NA
PDGFA	XM_036164870.1	7.49 × 10^−2^	5.48 × 10^−1^	ns	5.93 × 10^−7^	NA
PDGFD	NM_001357398.1	8.50 × 10^−2^	6.15 × 10^−1^	ns	8.91 × 10^−10^	TG
PENK	NM_001348209.1	4.59 × 10^−2^	6.85 × 10^−2^	NTG	1.78 × 10^−22^	NA
TAC4	NM_053093.2	8.18 × 10^−5^	6.98 × 10^−3^	NTG	4.30 × 10^−4^	TG
Receptor						
ADCYAP1R1	NM_001025372.2	1.08 × 10^−3^	4.32 × 10^−3^	NTG	1.03 × 10^−19^	NA
AVPR1B	NM_011924.2	7.57 × 10^−2^	9.56 × 10^−1^	ns	3.69 × 10^−3^	TG
NPFFR2	XM_017320589.3	9.61 × 10^−2^	7.87 × 10^−1^	ns	3.22 × 10^−3^	NA
NPR3	XM_030248376.1	9.71 × 10^−2^	9.06 × 10^−1^	ns	2.92 × 10^−3^	TG
OPRD1	NM_013622.3	7.03 × 10^−4^	6.78 × 10^−1^	ns	7.39 × 10^−4^	NA
TACR1	XM_006505865.4	6.10 × 10^−2^	2.83 × 10^−1^	ns	1.49 × 10^−18^	NA

^1^ FDR-adjusted *p*-value; ^2^ Dif: direction of change; NTG = nitroglycerin-elicited hyperalgesia overexpressed compared to control; Con = nitroglycerin-elicited hyperalgesia underexpressed compared to control; NA = nucleus accumbens overexpressed trigeminal ganglia; TG = nucleus accumbens underexpressed trigeminal ganglia; ns = non-significant difference.

**Table 2:** Neuropeptide prohormone and receptor transcript isoforms and genes that present significant (FDR-adjusted *p*-value < 0.05) effect of nitroglycerin-elicited hypersensitivity to pain and significant (FDR-adjusted *p*-value < 0.05) region difference.

Symbol	Accession	Treatment		Region	
		FC ^1^	PV ^2^	FC	PV
Prohormone					
APLN	NM_013912.4	1.43	3.77 × 10^−2^	7.38	1.79 × 10^−12^
CALCA	NM_007587.2	0.42	4.23 × 10^−2^	0.04	2.92 × 10^−49^
IGF1	NM_010512.5	2.51	1.90 × 10^−2^	0.13	1.32 × 10^−4^
IGF2	NM_010514.3	2.37	2.93 × 10^−2^	0.41	1.34 × 10^−2^
NMB	NM_001291280.1	0.76	1.90 × 10^−2^	0.04	1.95 × 10^−18^
NPFF	NM_018787.1	0.76	3.95 × 10^−2^	0.52	3.89 × 10^−6^
NPW	NM_001099664.2	0.11	1.84 × 10^−2^	0.05	1.52 × 10^−3^
PNOC	XM_006518684.4	5.33	2.93 × 10^−2^	7.44	3.63 × 10^−3^
SCG3	NM_009130.3	0.89	2.88 × 10^−2^	0.46	4.04 × 10^−14^
Receptor					
EDNRB	NM_007904.4	0.87	1.86 × 10^−2^	0.50	1.61 × 10^−12^
HCRTR1	NM_001357258.1	0.29	2.18 × 10^−3^	0.28	1.44 × 10^−3^

^1^ FC = fold change. Treatment fold change for nitroglycerin-elicited hyperalgesia compared to control; region fold change for nucleus accumbens compared to trigeminal ganglia; ^2^ PV = FDR-adjusted *p*-value.

**Table 3:** Neuropeptide prohormone and receptor transcript isoforms and genes presenting a significant (FDR-adjusted *p*-value < 0.05) effect of nitroglycerin-elicited hypersensitivity to pain, irrespective of region.

Symbol	Accession	FC ^1^	FDR PV ^2^
Prohormone			
CALCA	NM_001289444.1	0.56	2.06 × 10^−2^
CALCA	XM_011241660.4	0.66	2.42 × 10^−2^
CORT	NM_007745.4	1.44	2.26 × 10^−6^
IGF2	NM_010514.3	2.37	2.93 × 10^−2^
NPPB	NM_008726.6	0.84	4.08 × 10^−3^
PDGFA	XM_011240971.4	2.10	2.41 × 10^−2^
PDGFA	XM_030254199.2	0.56	2.17 × 10^−5^
PMCH	NM_029971.2	1.39	1.93 × 10^−3^
VIP	NM_011702.3	0.33	5.81 × 10^−3^
Receptor			
CALCR	NM_007588.2	0.88	3.81 × 10^−2^
GIPR	NM_001080815.1	0.19	3.15 × 10^−3^
GIPR	XR_004934120.1	0.68	1.43 × 10^−4^
NPY5R	NM_016708.3	1.72	3.62 × 10^−2^
PTH1R	NM_001083936.1	0.61	4.19 × 10^−2^
PTH2R	XM_006495843.4	0.16	4.12 × 10^−2^
PTH2R	XM_017319687.1	1.44	2.45 × 10^−4^

^1^ FC = fold change. Treatment fold change for nitroglycerin-elicited hyperalgesia compared to control; ^2^ PV = FDR-adjusted *p*-value.

**Table 4:** Frequency of significant (FDR-adjusted *p*-value < 0.05) differentially expressed neuropeptide prohormone and receptor transcript isoforms between nucleus accumbens and trigeminal ganglia.

Symbol	Number of Transcripts ^1^					FC ^2^
	Tot	M	NS	O	U	
Prohormone						
ADCYAP1	8	0	4	0	4	0.1
CALCA	7	1	3	0	3	0.0
CCK	3	0	0	3	0	35.3
IGF1	20	9	8	1	2	0.4
IGF2	8	2	3	0	3	0.4
NUCB2	4	0	1	3	0	2.8
PDGFA	8	1	4	1	2	1.0
PNOC	4	0	1	3	0	9.8
SCG3	4	1	0	0	3	0.5
TOR2A	8	1	4	3	0	2.5
VGF	12	0	9	3	0	9.0
Receptor						
ADCYAP1R1	12	3	4	5	0	8.8
CRHR1	9	2	4	3	0	3.4
GIPR	7	0	4	3	0	5.2
NPR2	4	0	0	1	3	0.5
NPR3	8	0	5	0	3	0.3
NTSR2	4	0	1	3	0	8.4
OPRL1	20	5	12	2	1	2.3
PDGFRB	4	0	0	0	4	0.5
SCTR	15	7	5	2	1	2.4
SSTR3	4	0	1	3	0	7.4

^1^ Tot = total number of annotated transcript isoforms; M = number of transcript isoforms not analyzed; NS = number of transcript isoforms not differentially expressed (FDR-adjusted *p*-value > 0.05); O = number of significantly overexpressed transcript isoforms in the nucleus accumbens compared to trigeminal ganglia; U = number of significantly underexpressed transcript isoforms in the nucleus accumbens compared to trigeminal ganglia; ^2^ FC = fold change in the nucleus accumbens compared to trigeminal ganglia averaged across significantly differentially expressed transcript isoforms.

**Table 5:** Number of neuropeptide prohormone and receptor genes presenting significant (FDR-adjusted *p*-value < 0.1) alternative spliced events across treatments (NTG = nitroglycerin-elicited hyperalgesia and CON = control).

Symbol	AS ^1^		Accession ^2^		Effect ^3^		
	T	L	Short	Long	C	FDR	D
Prohormone							
CALCA	A5	5′	NM_001033954.3	NM_001289444.1	NAc	0.099	0.08
VGF	MX	5′	XM_030254622.1	XM_006504434.3	TG	0.044	0.41
Receptor							
ADCYAP1R1	SE	E	XM_030255114.1	NM_007407.4	TG	0.003	0.29
		E	XM_030255114.1	XM_011241150.1	TG	0.033	0.22
CRHR2	A3	E	NM_009953.4	NM_001288618.1	Joint	0.001	0.10
					TG	0.019	0.12
IGF1R	A3	E	NM_010513.2	XM_006540641.5	NAc	0.028	−0.08

^1^ AS = alternative splicing; T = type of alternative splicing: A3 = alternative 3′ splice site event, A5 = alternative 5′ splice site event, MX = mutually exclusive exons event, SE = skipped exon event. L = location of splice event: 5′ = 5′ untranslated region; E = exon. ^2^ Accession: Example accession number of a transcript isoform exhibiting the short or long form of the splicing event. ^3^ Effect: C = comparison: NAc = nucleus accumbens only; TG = trigeminal ganglia only. Joint combined across nucleus accumbens and trigeminal ganglia. FDR = false discovery rate adjusted *p*-value. D = difference between the percentage of read counts adjusted for effective isoform exon lengths between NTG and control samples.

**Table 6:** Neuropeptide prohormone and receptor genes presenting significant (FDR-adjusted *p*-value < 0.1) alternative spliced events between nucleus accumbens (NAc) and trigeminal ganglia (TG).

Symbol	Alternative Splice Event ^1^										Location and Effect ^2^
	A3SS		A5SS		MutX		RetIntron		SkipExon		
	N	D	N	D	N	D	N	D	N	D	
Prohormone											
NUCB2									1	−0.04	Coding Un
SCG2							1	0.06			Coding Un
SCG3									2	−0.04	Coding Un
TAC1					1	−0.07			1	0.06	Coding Kn
VGF			1	0.08							5′ UTR
Receptor											
ADCYAP1R1	1	−0.06			1	0.01					Coding Kn
CRHR1									2	0.06	Coding
EDNRB							1	−0.05			3′ UTR
INSR									1	−0.07	Coding Un
NPR2					1	0.24			1	−0.46	Coding Kn
NPY1R									1	0.26	5′ UTR
OPRL1			1	0.17	2	0.34			5	0.09	N-terminal

^1^ A3SS = alternative 3′ splice site; A5SS = alternative 5′ splice site; MutX = mutually exclusive exon; RetIntron = retained intron; SkipExon = skipped exon; N = number of significant (FDR-adjusted *p*-value < 0.1) alternative spliced events; D = difference between percentage of read counts adjusted for effective isoform exon lengths between all nucleus accumbens and trigeminal ganglia samples. ^2^ 3′ UTR = alternative splicing event located in 3′ untranslated region; 5′ UTR = alternative splicing event located in 5′ untranslated region; Coding Kn = alternative splicing event involving the coding region of known transcript isoforms; Coding Un = alternative splicing event involving the coding region of uncharacterized transcript isoforms; N-terminal = alternative splicing event located N-terminal region including 5′ untranslated and coding regions.

## Data Availability

The data utilized in this study are openly available in the National Center for Biotechnology Information Gene Expression Omnibus (GEO) repository, experiment identifier GSE110194.

## Supplementary Materials

The following are available online at https://www.mdpi.com/article/10.3390/biomedicines10040877/s1, Table S1: Number of complete and partial neuropeptide prohormone and receptor sequences recovered using de novo assembly in the trigeminal ganglia (TG) and nucleus accumbens (NAc) of nitroglycerin-elicited hypersensitive (NTG) and control (CON) mice, Table S2: Detection of de novo neuropeptide genes across mouse treatment and region and corresponding identification among 10 peptidomic experiments.
