# Supplementary material for: Alternative Splicing of Neuropeptide Prohormone and Receptor Genes Associated with Pain Sensitivity Was Detected with Zero-Inflated Models
Source: Biomedicines. 2022 Apr 10;10(4):877. doi: 10.3390/biomedicines10040877 (PMC9031102; doi:10.3390/biomedicines10040877)
Supplement: Supplementary file 1 [file biomedicines-10-00877-s001.zip › biomedicines-1655398-supplementary.pdf]

Table S1. Number of complete and partial prohormone neuropeptide and receptor sequences recovered using de novo assembly in the nucleus accumbens (NAc) and trigeminal ganglia (TG) of nitroglycerin-elicited hypersensitive (NTG) and control (CON) mice.

NTG		CON		Prohormone	Receptor
NAc	TG	NAc	TG		
NF[1]	NF	CF	CF	23	9
			CP	1	1
		CP	CF	0	2
			CP	0	0
	NP	CF	CF	0	
			CP	10	10
			CZ	1	1
			NF		3
		CP	CF	0	
			CP	1	3
			CZ	0	0
			NF		0
	NZ	CF	CP	1	0
			CZ	0	1
		CP	CP	0	0
			CZ	0	2
NP	NF	CF	CF	1	2
			CP	0	0
		CP	CF	10	3
			CP	6	3
		CZ	CF	0	0
			CP	0	0
	NP	CF	CF	0	
			CP	2	1
			CZ	0	0
			NF		3
		CP	CF	0	
			CP	54	54
			CZ	3	1
			NF		3
		CZ	CF	0	
			CP	2	1
			CZ	0	1
			NF		0
	NZ	CF	CP	0	0
			CZ	0	0
		CP	CP	2	6
			CZ	11	34
		CZ	CP	0	0
			CZ	3	7
NZ	NF	CP	CF	0	0

		CP	0	1
	CZ	CF	0	0
		CP	0	0
NP	CP	CF	0	
		CP	7	5
		CZ	2	1
		NF		0
	CZ	CF	1	
		CP	4	13
		CZ	4	2
		NF		0
NZ	CP	CP	2	2
		CZ	0	10
	CZ	CP	1	0
		CZ	12	33

[1] NF: NTG sample with complete recovery of protein sequence; CF: Control sample with complete recovery of protein sequence; NP: NTG sample with partial recovery of protein sequence; CP: Control sample with partial recovery of protein sequence; NZ: NTG sample with no recovery of protein sequence; CZ: Control sample with no recovery of a protein sequence.

Table S2. Detection of de novo neuropeptide genes across mouse treatment and region and corresponding identification among 10 peptidomics experiments.

Identifier[1]		Count[2]				Region[3]														
Symbol	MGI	nN	nT	vN	vT	AM	DH	HA	HC	HT	NAc	PG	PC	RM	SCN	SON	ST	TH	TG	TN
ADCYAP1	105094	5	5	4	5		X	X	X	X	X	X			X	X	X			X
CARTPT	1351330	5	5	5	5	X	X	X	X	X	X	X			X	X	X			X
CCK	88297	5	5	5	5	X	X	X	X	X	X		X		X		X			X
CHGA	88394	5	5	5	5	X	X	X	X	X	X	X	X	X	X		X	X	X	X
CHGB	88395	5	5	5	5	X	X	X	X	X	X	X	X	X	X	X	X	X		X
GRP	95833	3	4	5	1	X	X	X	X	X	X				X		X	X		X
NPY	97374	5	4	5	5	X	X	X	X	X	X	X	X	X	X		X			X
NTS	1328351	5	5	5	5	X	X	X	X	X	X	X				X	X	X		X
PCSK1N	1353431	5	5	5	5	X	X	X	X	X	X	X	X	X	X	X	X	X	X	X
PDYN	97535	5	4	5	5	X	X	X	X	X	X		X	X	X	X	X	X		X
PENK	104629	5	5	5	5	X	X	X	X	X	X	X	X	X	X	X	X	X		X
PMCH	97629	5	5	5	2		X	X	X	X	X	X	X		X	X	X			X
PNOC	105308	5	5	5	3	X	X	X	X	X	X	X	X		X		X	X		
POMC	97742	5	5	5	5		X	X		X	X			X	X	X	X		X	X
SCG2	103033	5	5	5	5	X		X	X	X	X		X	X	X	X	X	X	X	X
SCG3	103032	5	5	5	5	X	X	X	X	X	X		X		X	X	X	X	X	X
SST	98326	5	5	5	5		X	X	X	X	X	X		X	X	X	X			X
TAC1	98474	5	5	5	5	X		X	X	X	X		X		X	X	X	X		
TAC3	98476	5	1	5	0	X	X	X	X	X	X		X		X		X	X		
TRH	98823	5	1	5	3	X	X	X	X	X	X	X		X	X	X	X			X
VGF	1343180	5	5	5	5	X	X	X	X	X	X	X	X	X	X		X	X	X	X

[1] Identifier: Symbol= ADCYAP1:adenylate cyclase activating polypeptide 1; CARTPT:CART prepropeptide; CCK:cholecystokinin; CHGA:chromogranin A; CHGB:chromogranin B; GRP:gastrin releasing peptide; NPY:neuropeptide Y; NTS:neurotensin; PCSK1N:proprotein convertase subtilisin/kexin type 1 inhibitor; PDYN:prodynorphin; PENK:preproenkephalin; PMCH:pro-melanin-concentrating hormone; PNOC:prepronociceptin; POMC:pro-opiomelanocortin-alpha; SCG2:secretogranin II; SCG3:secretogranin III; SST:somatostatin; TAC1:tachykinin 1; TAC3:Tachykinin 2/3; TRH:thyrotropin releasing hormone; VGF:VGF nerve growth factor inducible. MGI= Mouse Genome Informatics gene id suffix.

[2] Count: number samples with de novo matches in nitroglycerin group in nucleus accumbens (nN), nitroglycerin group in trigeminal ganglia (nT), control group in nucleus accumbens (vN), and control group in trigeminal ganglia (vT).

[3] Region: amygdala (AM) [1], Dorsal horn (DH) [2], habenula (HA) [3], hippocampus (HC) [1,4], hypothalamus (HT) [1,2,4,5], nucleus accumbens (NAc) [2,4], periacqueductal gray (PG) [2], prefrontal cortex (PC) [1], rostroventral medulla (RM) [2], suprachiasmatic nucleus (SCN) [6-8], supraoptic nucleus (SON) [9], striatum or dorsal striatum (ST) [1,4], thalamus (TH) [1], trigeminal ganglia (TG) [2], and trigeminal nucleus (TN) [2].

References

1. Zhang, X.; Che, F.Y.; Berezniuk, I.; Sonmez, K.; Toll, L.; Fricker, L.D. Peptidomics of Cpe(fat/fat) mouse brain regions: implications for neuropeptide processing. *J Neurochem* **2008**, *107*, 1596-1613, doi:10.1111/j.1471-4159.2008.05722.x.
2. Anapindi, K.D.B.; Yang, N.; Romanova, E.V.; Rubakhin, S.S.; Tipton, A.; Dripps, I.; Sheets, Z.; Sweedler, J.V.; Pradhan, A.A. PACAP and Other Neuropeptide Targets Link Chronic Migraine and Opioid-induced Hyperalgesia in Mouse Models. *Mol Cell Proteomics* **2019**, *18*, 2447-2458, doi:10.1074/mcp.RA119.001767.
3. Yang, N.; Anapindi, K.D.B.; Rubakhin, S.S.; Wei, P.; Yu, Q.; Li, L.; Kenny, P.J.; Sweedler, J.V. Neuropeptidomics of the Rat Habenular Nuclei. *J Proteome Res* **2018**, *17*, 1463-1473, doi:10.1021/acs.jproteome.7b00811.
4. Ye, H.; Wang, J.; Tian, Z.; Ma, F.; Dowell, J.; Bremer, Q.; Lu, G.; Baldo, B.; Li, L. Quantitative Mass Spectrometry Reveals Food Intake-Induced Neuropeptide Level Changes in Rat Brain: Functional Assessment of Selected Neuropeptides as Feeding Regulators. *Mol Cell Proteomics* **2017**, *16*, 1922-1937, doi:10.1074/mcp.RA117.000057.
5. Yang, N.; Anapindi, K.D.B.; Romanova, E.V.; Rubakhin, S.S.; Sweedler, J.V. Improved identification and quantitation of mature endogenous peptides in the rodent hypothalamus using a rapid conductive sample heating system. *Analyst* **2017**, *142*, 4476-4485, doi:10.1039/c7an01358b.
6. Lee, J.E.; Atkins, N.; Hatcher, N.G.; Zamdborg, L.; Gillette, M.U.; Sweedler, J.V.; Kelleher, N.L. Endogenous peptide discovery of the rat circadian clock: a focused study of the suprachiasmatic nucleus by ultrahigh performance tandem mass spectrometry. *Molecular & cellular proteomics: MCP* **2010**, *9*, 285-297, doi:10.1074/mcp.M900362-MCP200.
7. Lee, J.E.; Zamdborg, L.; Southey, B.R.; Atkins, N., Jr.; Mitchell, J.W.; Li, M.; Gillette, M.U.; Kelleher, N.L.; Sweedler, J.V. Quantitative peptidomics for discovery of circadian-related peptides from the rat suprachiasmatic nucleus. *J Proteome Res* **2013**, *12*, 585-593, doi:10.1021/pr300605p.
8. Southey, B.R.; Lee, J.E.; Zamdborg, L.; Atkins, N., Jr.; Mitchell, J.W.; Li, M.; Gillette, M.U.; Kelleher, N.L.; Sweedler, J.V. Comparing label-free quantitative peptidomics approaches to characterize diurnal variation of peptides in the rat suprachiasmatic nucleus. *Anal Chem* **2014**, *86*, 443-452, doi:10.1021/ac4023378.
9. Bora, A.; Annangudi, S.P.; Millet, L.J.; Rubakhin, S.S.; Forbes, A.J.; Kelleher, N.L.; Gillette, M.U.; Sweedler, J.V. Neuropeptidomics of the supraoptic rat nucleus. *Journal of Proteome Research* **2008**, *7*, 4992-5003, doi:10.1021/pr800394e.
